# The viscosity iterative algorithms for the implicit midpoint rule of nonexpansive mappings in uniformly smooth Banach spaces

**DOI:** 10.1186/s13660-017-1426-8

**Published:** 2017-06-28

**Authors:** Ping Luo, Gang Cai, Yekini Shehu

**Affiliations:** 1 School of Mathematics Science, Chongqing Normal University, Chongqing, 401331 China; 2 Department of Mathematics, University of Nigeria, Nsukka, Nigeria

**Keywords:** 49H09, 47H10, 47J20, strong convergence, nonexpansive mapping, implicit midpoint rule, uniformly smooth Banach space

## Abstract

The aim of this paper is to introduce a viscosity iterative algorithm for the implicit midpoint rule of nonexpansive mappings in uniformly smooth spaces. Under some appropriate conditions on the parameters, we prove some strong convergence theorems. As applications, we apply our main results to solving fixed point problems of strict pseudocontractive mappings, variational inequality problems in Banach spaces and equilibrium problems in Hilbert spaces. Finally, we give some numerical examples for supporting our main results.

## Introduction

Throughout this paper, we assume that *E* and $E^{*}$ is a real Banach space and the dual space of *E*, respectively. Let *T* be a mapping from *C* into itself, where *C* is a subset of *E*. We denoted by $F(T)$ the set of fixed points of *T*. It is well known that the duality mapping $J:E\rightarrow2^{E^{*}}$ is defined by

$$J(x)= \bigl\{ x^{*}\in E^{*}: \bigl\langle x,x^{*} \bigr\rangle = \Vert x \Vert ^{2}, \bigl\Vert x^{*} \bigr\Vert = \Vert x \Vert \bigr\} ,\quad \forall x\in E. $$

When *J* is single-valued, we denote it by *j*. We notice that if *E* is a Hilbert space, then *J* is the identity mapping and if *E* is smooth, then *J* is single-valued.

Now we recall some basic concepts and facts appeared in [1]. A mapping $f: C\rightarrow C $ is said to be a contraction, if there exists a constant $\alpha\in[0,1)$ satisfying

$$\bigl\Vert f(x)-f(y) \bigr\Vert \leq\alpha \Vert x-y \Vert ,\quad \forall x,y\in C. $$

We use $\Pi_{C}$ to denote the collection of all contractions from *C* into itself.

A mapping $T:C\rightarrow C$ is said to be nonexpansive if

1.1 $$ \Vert Tx-Ty \Vert \leq \Vert x-y \Vert ,\quad\forall x,y\in C. $$

Let $\rho_{E}:[0,\infty)\rightarrow[0,\infty)$ be defined by

$$\rho_{E}(t)=\sup \biggl\{ \frac{1}{2}\bigl( \Vert x+y \Vert + \Vert x-y \Vert \bigr)-1: x\in S(E), \Vert y \Vert \leq t \biggr\} , $$

which is called the modulus of smoothness of *E*. We say that Banach space *E* is uniformly smooth if $\dfrac{\rho_{E}(t)}{t}\rightarrow0$ as $t\rightarrow0$. It is well known that typical example of uniformly smooth Banach spaces is $L^{p}$, here $p>1$. Moreover, we say that Banach space *E* is *q*-uniformly smooth, if there exists a fixed constant $c>0$ such that $\rho_{E}(t)\leq ct^{q}$.

Recently, viscosity iterative algorithms for finding a common element of the set of fixed points for nonlinear operators and the set of solutions of variational inequality problems have been investigated by many authors; see [1–7] and the references therein. For example, Xu [1] introduced the explicit viscosity method for nonexpansive mappings:

1.2 $$ x_{n+1}=\alpha_{n}f(x_{n})+(1- \alpha_{n})Tx_{n},\quad n\geq0, $$

where $\{\alpha_{n} \}$ is a sequence in $(0,1)$ and $f\in \Pi_{C}$. Under some suitable conditions on $\{\alpha_{n} \}$, he proved that the sequence $\{x_{n} \}$ generated by (1.2) converges strongly to a fixed point *q* of *T* in Hilbert spaces or uniformly smooth Banach spaces, which also solves the variational inequality:

1.3 $$ \bigl\langle (I-f)q,x-q \bigr\rangle \geq0,\quad x\in F(T). $$

On the other hand, the implicit midpoint rule is a powerful method for solving ordinary differential equations; see [8–10] and the references therein. Recently, Xu *et al.* [11] applied the viscosity technique to the implicit midpoint rule for a nonexpansive mapping. Precisely, they considered the following viscosity implicit midpoint rule:

1.4 $$ x_{n+1}=\alpha_{n}f(x_{n})+(1- \alpha_{n})T\biggl(\frac{x_{n}+x_{n+1}}{2}\biggr),\quad n\geq 0. $$

They proved that the sequence generated by (1.4) converges strongly to a fixed point of *T*, which also solves the variational inequality (1.3) in Hilbert space. The following problems arise:

Question 1. Can we extend and improve the main results of Xu *et al.* [11] from Hilbert space to general Banach space? For example we might consider a uniformly smooth Banach space.

Question 2. We note that the proof of step 6 in Theorem 3.1 of [11] is very complicated. Can we simplify it?

In this paper, we give the affirmative answers to the above two questions. More precisely, we investigate the viscosity iterative algorithm (1.4) for the implicit midpoint rule of a nonexpansive mapping in a real uniformly smooth space. Under some suitable conditions on the parameters, we prove some strong convergence theorems. We also apply our main results to solve fixed point problems for strict pseudocontractive mappings, variational inequality problems in Banach spaces and equilibrium problems in Hilbert spaces.

## Preliminaries

The following lemmas are fundamental in the proof of our main results of this section.

### Lemma 2.1

[1]

*Assume* $\{a_{n} \}$ *is a sequence of nonnegative real numbers such that*

$$a_{n+1}\leq(1-\alpha_{n})a_{n}+ \delta_{n},\quad n\geq0, $$

*where* $\{\alpha_{n} \}$ *is a sequence in* $(0,1)$ *and* $\{ \delta_{n} \}$ *is a sequence in* $\mathbb{R}$ *such that*

(i) $\sum_{n=0}^{\infty}\alpha_{n}=\infty$, *and*
(ii) *either* $\limsup_{n\rightarrow\infty}\frac{\delta_{n}}{\alpha _{n}}\leq0$ *or* $\sum_{n=1}^{\infty} \vert \delta_{n} \vert <\infty$.

*Then* $\lim_{n\rightarrow\infty}a_{n}=0$.

### Lemma 2.2

[1]

*Let* *E* *be a uniformly smooth Banach space*, *C* *be a closed convex subset of* *E*, $T:C\rightarrow C$ *be a nonexpansive mapping with* $F(T)\neq\emptyset$ *and let* $f\in\Pi_{C}$. *Then the sequence* $\{x_{t} \}$ *defined by* $x_{t}=tf(x_{t})+(1-t)Tx_{t} $ *converges strongly to a point in* $F(T)$. *If we define a mapping* $Q:\Pi_{C}\rightarrow F(T)$ *by* $Q(f):=\lim_{t\rightarrow0}x_{t}$, $\forall f\in\Pi_{C}$. *Then* $Q(f)$ *solves the following variational inequality*:

$$\bigl\langle (I-f)Q(f),j\bigl(Q(f)-p\bigr) \bigr\rangle \leq0,\quad\forall f\in \Pi_{C}, p\in F(T). $$

### Lemma 2.3

[3]

*Let* *C* *be a nonempty closed convex subset of a real Banach space* *E* *which has uniformly Gâteaux differentiable norm*, *and* $T:C\rightarrow C$ *be a nonexpansive mapping with* $F(T)\neq\emptyset $. *Assume that* $\{z_{t} \}$ *strongly converges to a fixed point* *z* *of* *T* *as* $t\rightarrow0$, *where* $\{z_{t} \}$ *is defined by* $z_{t}=tf(z_{t})+(1-t)Tz_{t} $. *Suppose* $\{x_{n} \}\subset C$ *is bounded and* $\lim_{n\rightarrow\infty} \Vert x_{n}-Tx_{n} \Vert =0$. *Then*

$$\limsup_{n\rightarrow\infty} \bigl\langle f(z)-z,j(x_{n+1}-z) \bigr\rangle \leq0. $$

## Main results

### Theorem 3.1

*Let* *C* *be a closed convex subset of a uniformly smooth Banach space* *E*. *Let* $T:C\rightarrow C$ *be a nonexpansive mapping with* $F(T)\neq \emptyset$, *and* $f:C\rightarrow C$ *a contraction with coefficient* $\alpha\in[0,1)$. *Let* $\{x_{n} \}$ *be a sequence generated by the following viscosity implicit midpoint rule*:

3.1 $$ x_{n+1}=\alpha_{n}f(x_{n})+(1- \alpha_{n})T\biggl(\frac{x_{n}+x_{n+1}}{2}\biggr),\quad n\geq 0, $$

*where* $\{\alpha_{n} \}$ *is a sequence in* $(0,1)$ *such that*:

(i) $\lim_{n\rightarrow\infty}\alpha_{n}=0$,
(ii) $\sum_{n=0}^{\infty}\alpha_{n}=\infty$,
(iii) *either* $\sum_{n=0}^{\infty} \vert \alpha_{n+1}-\alpha _{n} \vert <\infty$ *or* $\lim_{n\rightarrow\infty}\frac{\alpha _{n+1}}{\alpha_{n}}=1$.

*Then* $\{x_{n} \}$ *converges strongly to a fixed point* *q* *of* *T*, *which also solve the following variational inequality*:

3.2 $$ \bigl\langle (I-f)q,j(x-q) \bigr\rangle \geq0,\quad x\in F(T). $$

### Proof

Using similar argument used in the proof of Theorem 3.1 of [11], we can find that the sequence $\{x_{n} \}$ is bounded and

3.3 $$ \Vert x_{n+1}-x_{n} \Vert \rightarrow0,\qquad \Vert x_{n}-Tx_{n} \Vert \rightarrow0,\quad\text{as }n\rightarrow \infty . $$

We omit the details. Let $\{x_{t} \}$ be a sequence defined by $x_{t}=tf(x_{t})+(1-t)Tx_{t}$, then it follows from Lemma 2.2 that $\{ x_{t} \}$ converges strongly to a fixed point *q* of *T*, which solves the variational inequality:

$$\bigl\langle (I-f)q,j(x-q) \bigr\rangle \geq0,\quad x\in F(T). $$

By (3.3) and Lemma 2.3, we have

3.4 $$ \limsup_{n\rightarrow\infty} \bigl\langle f(q)-q,j(x_{n+1}-q) \bigr\rangle \leq0. $$

Finally, we prove that $x_{n}\rightarrow q$ as $n\rightarrow\infty$. In fact, we observe

$$\begin{aligned} &{\Vert x_{n+1}-q \Vert ^{2}} \\ &{\quad = \biggl\Vert (1-\alpha_{n}) \biggl(T\biggl(\frac{x_{n}+x_{n+1}}{2} \biggr)-q\biggr)+\alpha _{n}\bigl(f(x_{n})-q\bigr) \biggr\Vert ^{2}} \\ &{\quad =(1-\alpha_{n}) \biggl\langle T\biggl(\frac {x_{n}+x_{n+1}}{2} \biggr)-q,j(x_{n+1}-q) \biggr\rangle +\alpha _{n} \bigl\langle f(x_{n})-q,j(x_{n+1}-q) \bigr\rangle } \\ &{\quad \leq\frac{1-\alpha_{n}}{2}\bigl( \Vert x_{n}-q \Vert + \Vert x_{n+1}-q \Vert \bigr) \Vert x_{n+1}-q \Vert +\alpha _{n}\alpha \Vert x_{n}-q \Vert \Vert x_{n+1}-q \Vert } \\ &{\qquad{} +\alpha_{n} \bigl\langle f(q)-q,j(x_{n+1}-q) \bigr\rangle } \\ &{\quad =\frac{1-\alpha_{n}+2\alpha_{n}\alpha}{2} \Vert x_{n}-q \Vert \Vert x_{n+1}-q \Vert +\frac{1-\alpha_{n}}{2} \Vert x_{n+1}-q \Vert ^{2}+\alpha_{n} \bigl\langle f(q)-q,j(x_{n+1}-q) \bigr\rangle ,} \end{aligned}$$

which implies

$$\frac{1+\alpha_{n}}{2} \Vert x_{n+1}-q \Vert ^{2}\leq \frac {1-\alpha_{n}+2\alpha_{n}\alpha}{4}\bigl( \Vert x_{n}-q \Vert ^{2}+ \Vert x_{n+1}-q \Vert ^{2}\bigr)+\alpha_{n} \bigl\langle f(q)-q,j(x_{n+1}-q) \bigr\rangle . $$

Thus we obtain

$$\frac{1+3\alpha_{n}-2\alpha_{n}\alpha}{4} \Vert x_{n+1}-q \Vert ^{2}\leq \frac{1-\alpha_{n}+2\alpha_{n}\alpha}{4} \Vert x_{n}-q \Vert ^{2}+ \alpha_{n} \bigl\langle f(q)-q,j(x_{n+1}-q) \bigr\rangle . $$

This implies

3.5 $$\begin{aligned} &{\Vert x_{n+1}-q \Vert ^{2}} \\ &{\quad \leq\frac{1-\alpha_{n}+2\alpha_{n}\alpha}{1+3\alpha_{n}-2\alpha _{n}\alpha} \Vert x_{n}-q \Vert ^{2}+ \frac{4\alpha_{n}}{1+3\alpha _{n}-2\alpha_{n}\alpha} \bigl\langle f(q)-q,j(x_{n+1}-q) \bigr\rangle } \\ &{\quad =\biggl[1-\frac{4\alpha_{n}(1-\alpha)}{1+\alpha_{n}+2\alpha_{n}(1-\alpha )}\biggr] \Vert x_{n}-q \Vert ^{2}} \\ &{\qquad{} +\frac{4\alpha_{n}(1-\alpha )}{1+\alpha_{n}+2\alpha_{n}(1-\alpha)}\frac{ \langle f(q)-q,j(x_{n+1}-q) \rangle}{1-\alpha}.} \end{aligned}$$

We note

$$\frac{4\alpha_{n}(1-\alpha)}{1+\alpha_{n}+2\alpha_{n}(1-\alpha)}>\frac {4(1-\alpha)}{4-2\alpha}\alpha_{n}. $$

Apply Lemma 2.1 to (3.5), we have $x_{n}\rightarrow q$ as $n\rightarrow \infty$. This finishes the proof. □

It is well known that Hilbert space is uniformly smooth, then we obtain the main results of [11].

### Corollary 3.2

*Let* *C* *be a closed convex subset of a Hilbert space* *H*, $T:C\rightarrow C$ *a nonexpansive mapping with* $F(T)\neq\emptyset$, *and* $f:C\rightarrow C$ *a contraction with coefficient* $\alpha\in [0,1)$. *Let* $\{x_{n} \}$ *be generated by the following viscosity implicit midpoint rule*:

$$x_{n+1}=\alpha_{n}f(x_{n})+(1- \alpha_{n})T\biggl(\frac{x_{n}+x_{n+1}}{2}\biggr),\quad n\geq0, $$

*where* $\{\alpha_{n} \}$ *is a sequence in* $(0,1)$ *satisfying*:

(i) $\lim_{n\rightarrow\infty}\alpha_{n}=0$,
(ii) $\sum_{n=0}^{\infty}\alpha_{n}=\infty$,
(iii) *either* $\sum_{n=0}^{\infty} \vert \alpha_{n+1}-\alpha _{n} \vert <\infty$ *or* $\lim_{n\rightarrow\infty}\frac{\alpha _{n+1}}{\alpha_{n}}=1$.

*Then* $\{x_{n} \}$ *converges strongly to a fixed point* *q* *of* *T*, *which is also the unique solution of the following variational inequality*:

$$\bigl\langle (I-f)q,x-q \bigr\rangle \geq0,\quad x\in F(T). $$

## Applications

(I) Application to fixed point problems for strict pseudocontractive mappings.

We say that a mapping $T:C\rightarrow C$ is *λ*-strict pseudocontractive if there exists a fixed constant $\lambda\in(0,1)$ such that

4.1 $$ \bigl\langle Tx-Ty,j(x-y) \bigr\rangle \leq \Vert x-y \Vert ^{2}- \lambda \bigl\Vert (I-T)x-(I-T)y \bigr\Vert ^{2}, $$

for some $j(x-y)\in J(x-y)$ and for every $x,y\in C$. A simple computation shows that (4.1) is equivalent to the following inequality:

4.2 $$ \bigl\langle (I-T)x-(I-T)y,j(x-y) \bigr\rangle \geq\lambda \bigl\Vert (I-T)x-(I-T)y \bigr\Vert ^{2} $$

for some $j(x-y)\in J(x-y)$ and for every $x,y\in C$.

Now we give a relationship between strict pseudocontractive mapping and nonexpansive mapping.

### Lemma 4.1

[12]

*Let* *C* *be a nonempty closed convex subset of a real* 2-*uniformly smooth Banach space* *E* *and* $T:C\rightarrow C$ *be a* *λ*-*strict pseudocontractive mapping*. *For* $\alpha\in(0,1)$, *we define* $T_{\alpha}x:=(1-\alpha)x+\alpha Tx$. *Then*, *as* $\alpha\in(0,\frac{\lambda }{K^{2}}]$, *where* *K* *is the* 2-*uniformly smooth constant*. *Then* $T_{\alpha}: C\rightarrow C$ *is nonexpansive such that* $F(T_{\alpha})=F(T)$.

Using Theorem 3.1 and Lemma 4.1, we obtain the following results.

### Theorem 4.1

*Let* *C* *be a closed convex subset of a uniformly smooth Banach space* *E*. *Let* $T:C\rightarrow C$ *a* *λ*-*pseudocontractive mapping with* $F(T)\neq\emptyset$, *and* $f:C\rightarrow C$ *a contraction with coefficient* $\alpha\in[0,1)$. *Let* $\{x_{n} \}$ *be a sequence generated by the viscosity implicit midpoint rule*:

4.3 $$ x_{n+1}=\alpha_{n}f(x_{n})+(1- \alpha_{n})T_{\delta}\biggl(\frac {x_{n}+x_{n+1}}{2}\biggr),\quad n \geq0, $$

*where* $T_{\delta}$ *is a mapping from* *C* *into itself defined by* $T_{\delta}x:=(1-\delta)x+\delta Tx$, $x\in C$, $\delta\in(0,\frac {\lambda}{K^{2}}]$. *Assume that* $\{\alpha_{n} \}$ *is a sequence in* $(0,1)$ *such that*:

(i) $\lim_{n\rightarrow\infty}\alpha_{n}=0$,
(ii) $\sum_{n=0}^{\infty}\alpha_{n}=\infty$,
(iii) *either* $\sum_{n=0}^{\infty} \vert \alpha_{n+1}-\alpha _{n} \vert <\infty$ *or* $\lim_{n\rightarrow\infty}\frac{\alpha _{n+1}}{\alpha_{n}}=1$.

*Then* $\{x_{n} \}$ *converges strongly to a fixed point* *q* *of* *T*, *which also solve the variational inequality*:

$$\bigl\langle (I-f)q,j(x-q) \bigr\rangle \geq0,\quad x\in F(T). $$

(II) Application to variational inequality problems in Banach spaces.

Let *C* be a nonempty closed convex subset of a Hilbert space *H* and let $A:C\rightarrow H$ be a nonlinear mapping. It is well known that the classical variational inequality is to find $x^{*}$ such that

4.4 $$ \bigl\langle Ax^{*},x-x^{*} \bigr\rangle \geq0,\quad \forall x\in C. $$

We denoted by $\mathrm{VI}(A,C)$ the set of solutions of (4.4).

Recently, Ceng *et al.* [13] considered the problem of finding $(x^{*},y^{*})\in C\times C$ satisfying

4.5 $$ \textstyle\begin{cases} \langle\lambda Ay^{*}+x^{*}-y^{*},x-x^{*} \rangle\geq0, & \forall x\in C,\\ \langle\mu Bx^{*}+y^{*}-x^{*},x-y^{*} \rangle\geq0, & \forall x\in C, \end{cases} $$

which is called a general system of variational inequalities, where $A, B:C\rightarrow H$ are two nonlinear mappings, $\lambda>0$ and $\mu>0$ are two constants. Precisely, they introduced a relaxed extragradient method for finding a common element of the set of fixed points of a nonexpansive mapping and the set of solutions of variational inequality problem (4.5) in a real Hilbert space.

Now we consider the problem of finding $(x^{*},y^{*})\in C\times C$ satisfying

4.6 $$ \textstyle\begin{cases} \langle\lambda Ay^{*}+x^{*}-y^{*},j(x-x^{*}) \rangle\geq0,& \forall x\in C,\\ \langle\mu Bx^{*}+y^{*}-x^{*},j(x-y^{*}) \rangle\geq0,& \forall x\in C. \end{cases} $$

Problem (4.6) is called the system of general variational inequalities in a real Banach spaces. In particular, if *E* is a Hilbert space, then problem (4.6) becomes problem (4.5). So our problem (4.6) contains (4.5) as a special case.

Recall that a mapping $A:C\rightarrow E$ is called accretive if there exists some $j(x-y)\in J(x-y)$ such that

4.7 $$ \bigl\langle Ax-Ay,j(x-y) \bigr\rangle \geq0,\quad\forall x,y\in C. $$

A mapping $A:C\rightarrow E$ is said to be *α*-inverse-strongly accretive if there exist some $j(x-y)\in J(x-y)$ and a fixed constant $\alpha>0$ such that

4.8 $$ \bigl\langle Ax-Ay,j(x-y) \bigr\rangle \geq\alpha \Vert Ax-Ay \Vert ^{2},\quad\forall x,y\in C. $$

The following lemmas are very important for proving our main results.

### Lemma 4.2

[14]

*Let* *C* *be a nonempty closed convex subset of a real* 2-*uniformly smooth Banach space* *E*. *Let* $Q_{C}$ *be the sunny nonexpansive retraction from* *E* *onto* *C*. *Let the mappings* $A,B:C\rightarrow E$ *be* *α*-*inverse*-*strongly accretive and* *β*-*inverse*-*strongly accretive*, *respectively*. *Let* $G:C\rightarrow C$ *be a mapping defined by*

$$G(x)=Q_{C} \bigl[Q_{C}(x-\mu Bx)-\lambda AQ_{C}(x- \mu Bx) \bigr],\quad \forall x\in C. $$

*If* $0<\lambda\leq\frac{\alpha}{K^{2}}$ *and* $0<\mu\leq\frac{\beta}{K^{2}}$, *then* $G:C\rightarrow C$ *is nonexpansive*.

### Lemma 4.3

[14]

*Let* *C* *be a nonempty closed convex subset of a real* 2-*uniformly smooth Banach space* *E*. *Let* $Q_{C}$ *be the sunny nonexpansive retraction from* *E* *onto* *C*. *Let* $A,B:C\rightarrow E$ *be two nonlinear mappings*. *For given* $x^{*},y^{*}\in C$, $(x^{*},y^{*})$ *is a solution of problem* (4.6) *if and only if* $x^{*}=Q_{C}(y^{*}-\lambda Ay^{*})$ *where* $y^{*}=Q_{C}(x^{*}-\mu Bx^{*})$, *that is*, $x^{*}=Gx^{*}$, *where* *G* *is defined by Lemma* 4.2.

### Theorem 4.2

*Let* *C* *be a closed convex subset of a real* 2-*uniformly smooth Banach space* *E*, *let the mappings* $A,B:C\rightarrow E$ *be* *α*-*inverse*-*strongly accretive and* *β*-*inverse*-*strongly accretive with* $F(G)\neq\emptyset$, *where* $G:C\rightarrow C$ *is a mapping defined by Lemma* 4.2. *Let* $f:C\rightarrow C$ *be a contraction with coefficient* $\alpha\in[0,1)$. *Let* $\{x_{n} \}$ *be a sequence generated by the viscosity implicit midpoint rule*:

4.9 $$ \textstyle\begin{cases} x_{n+1}=\alpha_{n}f(x_{n})+(1-\alpha_{n})y_{n},\\ y_{n}=Q_{C}(u_{n}-\lambda Au_{n}),\\ u_{n}=Q_{C}(z_{n}-\mu Bz_{n}),\\ z_{n}=\frac{x_{n}+x_{n+1}}{2}, \end{cases} $$

*where* $0<\lambda\leq\frac{\alpha}{K^{2}}$, $0<\mu\leq\frac{\beta }{K^{2}}$. *Suppose that* $\{\alpha_{n} \}$ *is a sequence in* $(0,1)$ *satisfying*:

(i) $\lim_{n\rightarrow\infty}\alpha_{n}=0$,
(ii) $\sum_{n=0}^{\infty}\alpha_{n}=\infty$,
(iii) *either* $\sum_{n=0}^{\infty} \vert \alpha_{n+1}-\alpha _{n} \vert <\infty$ *or* $\lim_{n\rightarrow\infty}\frac{\alpha _{n+1}}{\alpha_{n}}=1$.

*Then* $\{x_{n} \}$ *converges strongly to a fixed point* *q* *of* *G*, *which is also the unique solution of the following variational inequality*:

$$\bigl\langle (I-f)q,j(x-q) \bigr\rangle \geq0,\quad x\in F(G). $$

### Proof

By Lemma 4.2, we see that *G* is nonexpansive. So we obtain the desired results by Theorem 3.1 immediately. □

(III) Application to equilibrium problems in Hilbert spaces.

Let $\phi:C\times C\rightarrow\mathbb{R}$ be a bifunction, where $\mathbb{R}$ is the set of real numbers. The equilibrium problem for the function *ϕ* is to find a point $x\in C$ satisfying

4.10 $$ \phi(x,y)\geq0\quad \mbox{for all } y\in C. $$

We denoted by $EP(\phi)$ the set of solutions of (4.10). This equilibrium problem contains variational inequality problem, optimization problem and the fixed point problem as its special cases (see Blum and Oettli [15] for more information).

For solving the equilibrium problem, we need to assume that the bifunction *ϕ* satisfies the following four conditions (see [15]):

1. $\phi(x,x)=0$ for all $x\in C$;
2. *ϕ* is monotone, that is, $\phi(x,y)+\phi(y,x)\leq0$ for all $x,y\in C$;
3. *ϕ* is upper-hemicontinuous, *i.e.*, for any $x,y,z\in C$
  $$\limsup_{t\rightarrow0^{+}}\phi\bigl(tz+(1-t)x,y\bigr)\leq\phi(x,y); $$
4. $\phi(x,\cdot)$ is convex and weakly lower semicontinuous for each $x\in C$.

In order to prove our main results, we need the following lemmas.

### Lemma 4.4

[15]

*Let* *C* *be a nonempty closed convex subset of* *H* *and let* *ϕ* *be a bifunction of* $C\times C$ *into* $\mathbb{R}$ *satisfying* (A1)-(A4). *Let* $r>0$ *and* $x\in H$. *Then there exists* $z\in C$ *such that*

$$\phi(z,y)+\frac{1}{r} \langle y-z,z-x \rangle\geq0\quad\textit{for all } y\in C. $$

### Lemma 4.5

[16]

*Assume that* $\phi:C\times C\rightarrow\mathbb {R}$ *satisfies* (A1)-(A4). *For* $r>0$ *and* $x\in H$, *define a mapping* $T_{r}:H\rightarrow C$ *as follows*:

$$T_{r}(x)= \biggl\{ z\in C:\phi(z,y)+\frac{1}{r} \langle y-z,z-x \rangle\geq0\ \forall y\in C \biggr\} $$

*for all* $z\in H$. *Then the following hold*:

1. $T_{r}$ *is single*-*valued*.
2. $T_{r}$ *is firmly nonexpansive*, *i*.*e*., *for any* $x,y\in H$, $\Vert T_{r}x-T_{r}y \Vert ^{2}\leq \langle T_{r}x-T_{r}y,x-y \rangle$.

*This implies that* $\Vert T_{r}x-T_{r}y \Vert \leq \Vert x-y \Vert $, ∀*x*, $y\in H$, *i*.*e*., $T_{r}$ *is a nonexpansive mapping*.

(3) $F(T_{r})=EP(\phi)$, $\forall r>0$.
(4) $EP(\phi)$ *is a closed and convex set*.

We say that a mapping *T* is attracting nonexpansive if it is nonexpansive and satisfies

$$\Vert Tx-p \Vert < \Vert x-p \Vert \quad\text{for all }x\notin F(T)\text{ and }p\in F(T). $$

The following lemma gives a relationship between a nonexpansive mapping and an attracting nonexpansive mapping.

### Lemma 4.6

[17]

*Suppose that* *E* *is strictly convex*, $T_{1}$ *an attracting nonexpansive and* $T_{2}$ *a nonexpansive mapping which have a common fixed point*. *Then we have* $F(T_{1}T_{2})=F(T_{2}T_{1})=F(T_{1})\cap F(T_{2})$.

### Theorem 4.3

*Let* *C* *be a nonempty closed convex subset of a real Hilbert space* *H*, $\phi:C\times C\rightarrow\mathbb {R}$ *be a bifunction satisfying the conditions* (A1)-(A4). *Let* $T:C\rightarrow C$ *be a nonexpansive mapping with* $F=F(T)\cap EP(\phi)\neq\emptyset$, *and* $f:C\rightarrow C$ *a contraction with coefficient* $\alpha\in[0,1)$. *Let* $\{x_{n} \}$ *be a sequence generated by the viscosity implicit midpoint rule*

4.11 $$ \textstyle\begin{cases} x_{n+1}=\alpha_{n}f(x_{n})+(1-\alpha_{n})Tu_{n},\\ u_{n}\in C\quad\textit{such that } \phi(u_{n},y)+\frac{1}{r} \langle y-u_{n},u_{n}-z_{n} \rangle\geq0,\ \forall y\in C,r>0, \\ z_{n}=\frac{x_{n}+x_{n+1}}{2},\quad n\geq0, \end{cases} $$

*where* $\{\alpha_{n} \}$ *is a sequence in* $(0,1)$ *such that*:

(i) $\lim_{n\rightarrow\infty}\alpha_{n}=0$,
(ii) $\sum_{n=0}^{\infty}\alpha_{n}=\infty$,
(iii) *either* $\sum_{n=0}^{\infty} \vert \alpha_{n+1}-\alpha _{n} \vert <\infty$ *or* $\lim_{n\rightarrow\infty}\frac{\alpha _{n+1}}{\alpha_{n}}=1$.

*Then* $\{x_{n} \}$ *converges strongly to a fixed point* *q* *of* *F*, *which also solves the following variational inequality*:

$$\bigl\langle (I-f)q,j(x-q) \bigr\rangle \geq0,\quad x\in F. $$

### Proof

We can rewrite (4.11) as

4.12 $$ x_{n+1}=\alpha_{n}f(x_{n})+(1- \alpha_{n})TT_{r}\biggl(\frac {x_{n}+x_{n+1}}{2} \biggr). $$

By Lemma 4.5, we know that $T_{r}$ is firmly nonexpansive. Furthermore, we can prove that $T_{r}$ is attracting nonexpansive. Indeed, for any $x\notin F(T_{r})$ and $y\in F(T_{r})$, we have

$$\begin{aligned} \Vert T_{r}x-T_{r}y \Vert ^{2}&\leq \langle T_{r}x-T_{r}y,x-y \rangle \\ &=\frac{1}{2}\bigl[ \Vert T_{r}x-T_{r}y \Vert ^{2}+ \Vert x-y \Vert ^{2}- \Vert T_{r}x-x \Vert ^{2}\bigr], \end{aligned}$$

which implies that

$$\begin{aligned} \Vert T_{r}x-T_{r}y \Vert ^{2}&\leq \Vert x-y \Vert ^{2}- \Vert T_{r}x-x \Vert ^{2} \\ &< \Vert x-y \Vert ^{2}. \end{aligned}$$

Therefore $T_{r}$ is attracting nonexpansive. By Lemma 4.6, we find that $F(TT_{r})=F(T)\cap F(T_{r})=F(T)\cap EP(\phi)=F$. So we easily get the desired results by Theorem 3.1. □

## Numerical examples

In the last section, we give two numerical examples where our main results may be applied.

### Example 5.1

Assume that $\mathbb{R}$ is a real line with the Euclidean norm. Let $f,T:\mathbb{R}\rightarrow\mathbb{R}$ be defined by $f(x)=\frac{1}{4}x$ and $Tx=\frac{1}{2}x$ for any $x\in\mathbb {R}$, respectively. It is easy to see that $F(T)= \{0 \}$. Let $\alpha _{n}=\frac{1}{n}$ for each $n\in\mathbb{N}$. Let $\{x_{n} \} $ be a sequence generated by (1.2) and $\{y_{n} \}$ be a sequence generated by (3.1), respectively. Then by Theorem 3.1 and Theorem 3.1 of [11], we find that $\{x_{n} \}$ and $\{y_{n} \}$ converge strongly to 0. We can rewrite (1.2) and (3.1) as follows:

5.1 $$\begin{aligned} &{x_{n+1}=\frac{2n-1}{4n}x_{n},} \end{aligned}$$

5.2 $$\begin{aligned} &{y_{n+1}=\frac{n}{3n+1}x_{n}.} \end{aligned}$$

**Figure 1:**
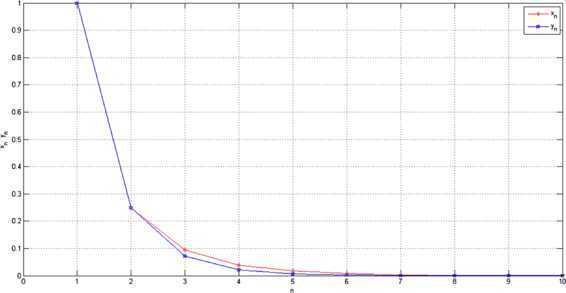
**Comparison.**

Choose $x_{1}=1$ and $y_{1}=1$ in (5.1) and (5.2), we get the following numerical results in Figure 1.

### Remark 5.2

By Figure 1, we know that $\{y_{n} \}$ converges to 0 more quickly than $\{x_{n} \}$. So the rate of convergence of viscosity implicit midpoint rule (3.1) is better than viscosity iterative algorithm (1.2).

### Example 5.3

Let $\langle \cdot,\cdot\rangle:\mathbb{R}^{3}\times\mathbb{R}^{3}\rightarrow \mathbb{R}$ be the inner product defined by

$$\langle\mathbf{x},\mathbf{y} \rangle=\mathbf{x}\cdot \mathbf{y}=x_{1} \cdot y_{1}+x_{2}\cdot y_{2}+x_{3}\cdot y_{3} $$

and let $\Vert \cdot \Vert :\mathbb{R}^{3}\rightarrow \mathbb{R}$ be the usual norm defined by $\Vert \mathbf{x} \Vert =\sqrt{x_{1}^{2}+y_{1}^{2}+z_{1}^{2}}$ for any $\mathbf{x}=(x_{1},x_{2},x_{3})$, $\mathbf{y}=(y_{1},y_{2},y_{3})\in\mathbb {R}^{3}$. For all $x\in\mathbb{R}$, let $T,f:\mathbb{R}^{3}\rightarrow \mathbb{R}^{3}$ be defined by $T\mathbf{x}=\frac{1}{3}\mathbf{x}$, and $f(\mathbf{x})=\frac {1}{3}\mathbf{x}$, respectively. Let $\alpha_{n}=\frac{1}{n}$ for each $n\in\mathbb{N}$. Assume that $\{x_{n} \}$ is a sequence generated by (3.1). We can see easily that $F(T)= \{0 \}$. Then $\{ \mathbf{x}_{n} \}$ converges strongly to 0. Moreover, we can rewrite (3.1) as follows:

5.3 $$ \mathbf{x}_{n+1}=\frac{3n+1}{15n+1}\mathbf{x}_{n}. $$

**Figure 2:**
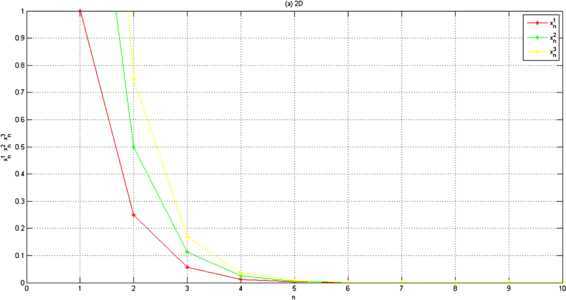
**Two dimension.**

**Figure 3:**
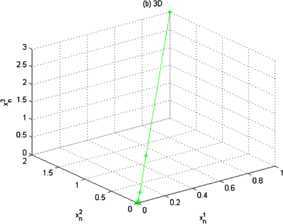
**Three dimension.**

Choose $\mathbf{x}_{1}=(1,2,3)$ in (5.3), we obtain the numerical results shown in Figure 2 and Figure 3.
